# Midventricular form of takotsubo cardiomyopathy as a recurrence 1 year after typical apical ballooning: a case report

**DOI:** 10.1186/1757-1626-1-331

**Published:** 2008-11-19

**Authors:** Oliver Koeth, Bernd Mark, Ralf Zahn, Uwe Zeymer

**Affiliations:** 1 Herzzentrum Ludwigshafen, Department of Cardiology, Ludwigshafen, Germany

## Abstract

Takotsubo cardiomyopathy was first described in Japan and is characterized by transient left ventricular apical ballooning in the absence of a significant coronary artery disease.

Caused by the clinical presentation including chest pain, electrocardiographic changes and elevated myocardial markers this syndrome is frequently misdiagnosed as an acute coronary syndrome. Recurrences of Takotsubo Cardiomyopathy, especially in variant regions of the left ventricle are rare

We describe a midventricular form of Takotsubo Cardiomyopathy as a recurrence 1 year after typical apical ballooning.

## Background

Takotsubo cardiomyopathy was first described in Japan and is characterized by transient left ventricular apical ballooning in the absence of a significant coronary artery disease [1].

Caused by the clinical presentation including chest pain, electrocardiographic changes and elevated myocardial markers this syndrome is frequently misdiagnosed as an acute coronary syndrome [2].

Takotsubo cardiomyopathy affects predominantly women and is often triggered by preceding emotional or physical stress [3]. However, the pathogenesis of the Takotsubo cardiomyopathy is still unknown. Catecholamines mediated cardio-toxicity provoked by emotional or physical stress, multivessel coronary vasospasm and abnormalities in coronary microvascular function have been proposed as possible explanations [2,3].

Takotsubo cardiomyopathy is named after the original Japanese octopus trap and is usually characterized by a left ventricular dysfunction with preserved basal function and apical akinesis.

We describe a midventricular form of Takotsubo Cardiomyopathy as a recurrence 1 year after typical apical ballooning.

## Case presentation

**Figure 1:**
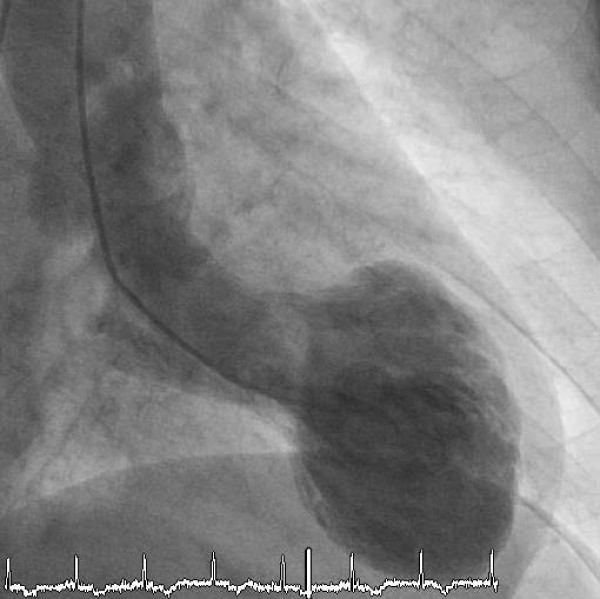
Typical apical ballooning (akinesia of midventricular and apical left ventricular segments and hyperkinesis of basal segments).

A 67- year old German female with a history of hypertension and Crohn's disease was admitted to our emergency department with chest pain. One year ago the patient was admitted with the same symptoms. A Takotsubo Cardiomyopathy was diagnosed and typical apical ballooning (akinesia of apical left ventricular segments and hyperkinesis of basal segments; Figure 1) was seen in the left ventricular angiogram. On admission she did not report about an obvious emotional stress situation (like news of an unexpected death of a relative or news of a catastrophic medical diagnosis) preceding chest pain. She was under chronic therapy with betablockers (Metoprolol 47,5 mg/od), ACE-inhibitors (Ramipril 2,5 mg/od) and aspirin (100 mg/od). Initially she had a pulse rate of 60 beats/min and a blood pressure of 120/80 mmHg. Her physical examination was essentially normal. Laboratory testing revealed elevated levels of Troponin T (0.13 ng/ml, [<0.03 ng/ml]) and creatinine kinase (208 U/l, [<145 U/l]]. Catecholamine plasma levels were not elevated.

The initial electrocardiogram showed sinus rhythm and T-inversions in the leads I, II, aVL, V1 and V2.

**Figure 2:**
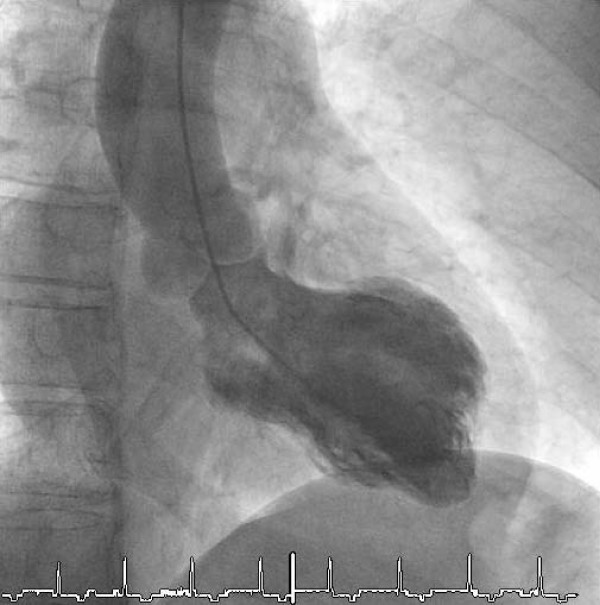
Isolated midventricular ballooning (akinesia anterolateral and diaphragmal).

An acute Non ST-elevation infarction was suspected based on the clinical presentation including chest pain, electrocardiographic changes and elevated myocardial markers. The patient received aspirin, clopidogrel und unfractionated heparin. Recent angiogram showed an isolated midventricular ballooning (akinesia anterolateral and diaphragmal; Figure 2) and an ejection fraction of 48 %. In both cases the coronary angiography showed a mild coronary artery disease with a 50 % stenosis in the left anterior descending. Contrast enhanced cardiac magnetic resonance imaging excluded myocardial necrosis as well as ischemia in the anterior wall.

Within one week wall motion abnormalities and ejection fraction fully recovered.

Her recovery was uneventful and she was doing well at discharge. She was discharged with a chronic medication including betablockers (Metoprolol 47,5 mg/td), ACE-inhibitors (Ramipril 2,5 mg/td), aspirin (100 mg/od) and statins (Simvastatin 40 mg/od).

## Discussion

The pathogenesis of Takotsubo cardiomyopathy is still unknown. Catecholamine mediated cardio toxicity provoked by emotional or physical stress has been proposed as explanation. A Takotsubo cardiomyopathy is associated with emotional stress in about 25 % of patients [2]. In the present case the patient did not report about an obvious emotional stress situation preceding chest pain. In addition catecholamine plasma levels were not elevated.

Most of the Takotsubo cardiomyopathies were observed in post- menopausal women and the most common clinical presentations are chest pain and dyspnoe [2]. Those findings are in line with the present case report.

Coronary angiography did not show a significant coronary disease. In addition contrast enhanced cardiac magnetic resonance imaging excluded myocardial necrosis as well as ischemia in the anterior wall. Therefore the diagnosis of a Takotsubo cardiomyopathy could be confirmed by excluding other reasons (myocarditis, embolic infarction) for acute left ventricular dysfunction.

In a minority of patients a different pattern with preserved apical contractile function and impaired midventricular contractility has been observed [4]. Additionally in a few patients recurrences of Takotsubo cardiomyopathy have been reported [5]. A recurrence in varying regions of the left ventricle, as observed in the present case, is rare [6]. Patients with a recurrence and/or atypical forms of Takotsubo cardiomyopathy do not differ in baseline characteristics, clinical presentation and in-hospital course compared to patients with typical Takotsubo cardiomyopathy.

In the present case wall motion abnormalities fully recovered and the ejection fraction resolved within one week. Recovery was uneventful and the patient was doing well at discharge. However, prognosis of patients with Takotsubo cardiomyopathy seems to be favorable, especially after an uncomplicated acute phase. Until now only a few cases of in-hospital mortality, cardiogenic shock, malignant ventricular arrhythmias and stroke due to thrombus formation in the left ventricle have been reported.

## Conclusion

Recurrences, especially in variant regions of the left ventricle are rare. Patients with recurrence and/or atypical forms of Takotsubo cardiomyopathy do not differ in baseline characteristics, clinical presentation and in-hospital course compared to patients with typical Takotsubo cardiomyopathy. Chronic therapy with betablockers (Metoprolol 47,5 mg/od) did not prevent a recurrence in this case, therefore therapy after the acute phase needs to be determinate.

## Consent

Written informed consent was obtained from the patient for publication of this case report and accompanying images. A copy of the written consent is available for review by the Editor-in-Chief of this journal.

## Competing interests

The authors declare that they have no competing interests.

## Authors' contributions

OK, BM, RZ and UZ treated the patient and were responsible for writing the paper and looking up the back ground references. RZ and UZ were responsible for over all coordination and final proof reading. All the above mentioned authors read and approved the final manuscript.
